# Multimorbidity: constellations of conditions across subgroups of midlife and older individuals, and related Medicare expenditures

**DOI:** 10.15256/joc.2017.7.91

**Published:** 2017-04-10

**Authors:** Siran M. Koroukian, Nicholas K. Schiltz, David F. Warner, Jiayang Sun, Kurt C. Stange, Charles W. Given, Avi Dor

**Affiliations:** ^1^Department of Epidemiology and Biostatistics, School of Medicine, Case Western Reserve University, Cleveland, OH, USA; ^2^Department of Sociology, University of Nebraska–Lincoln, Lincoln, NE, USA; ^3^Department of Family Medicine and Community Health, School of Medicine, Case Western Reserve University, Cleveland, OH, USA; ^4^Department of Family Medicine, Michigan State University, East Lansing, MI, USA; ^5^Tachtenberg School of Public Policy & Public Administration, George Washington University, Washington, DC, USA

**Keywords:** comorbidity, functional limitations, geriatric syndromes, multimorbidity, healthcare expenditures

## Abstract

**Introduction:**

The Department of Health and Human Services’ 2010 Strategic Framework on Multiple Chronic Conditions called for the identification of common constellations of conditions in older adults.

**Objectives:**

To analyze patterns of conditions constituting multimorbidity (CCMM) and expenditures in a US representative sample of midlife and older adults (50–64 and ≥65 years of age, respectively).

**Design:**

A cross-sectional study of the 2010 Health and Retirement Study (HRS; *n*=17,912). The following measures were used: (1) count and combinations of CCMM, including (i) chronic conditions (hypertension, arthritis, heart disease, lung disease, stroke, diabetes, cancer, and psychiatric conditions), (ii) functional limitations (upper body limitations, lower body limitations, strength limitations, limitations in activities of daily living, and limitations in instrumental activities of daily living), and (iii) geriatric syndromes (cognitive impairment, depressive symptoms, incontinence, visual impairment, hearing impairment, severe pain, and dizziness); and (2) annualized 2011 Medicare expenditures for HRS participants who were Medicare fee-for-service beneficiaries (*n*=5,677). Medicaid beneficiaries were also identified based on their self-reported insurance status.

**Results:**

No large representations of participants within specific CCMM categories were observed; however, functional limitations and geriatric syndromes were prominently present with higher CCMM counts. Among fee-for-service Medicare beneficiaries aged 50–64 years, 26.7% of the participants presented with ≥10 CCMM, but incurred 48% of the expenditure. In those aged ≥65 years, these percentages were 16.9% and 34.4%, respectively.

**Conclusion:**

Functional limitations and geriatric syndromes considerably add to the MM burden in midlife and older adults. This burden is much higher than previously reported.

## Introduction

In 2011, nearly two thirds of Medicare fee-for-service (FFS) beneficiaries had two or more chronic conditions, and 15% presented with six or more chronic conditions [1]. In addition, the average annual Medicare expenditures for beneficiaries with six or more chronic conditions was approximately threefold that of beneficiaries with four or five conditions (USD 31,543 vs. USD 11,628) [1].

The co-occurrence of multiple chronic conditions with functional limitations [2–10] and geriatric syndromes [11–14] has been well documented. In parallel, the shared risk factors between geriatric syndromes and functional dependence have also been described. In a 1995 study, Tinetti *et al*. [15] showed that decreased upper extremity impairment, decreased lower body impairment, sensory impairment, and affective impairment were predisposing factors for incontinence, falls, and functional dependence. Moreover, cognitive impairment has been shown to be associated with disability [16,17] and limitations in instrumental activities of daily living [18]. Extending the intricate association between geriatric syndromes and functional dependence to that of chronic conditions, Martinez *et al*. [19] demonstrated that both chronic obstructive pulmonary disease and mild cognitive impairment increased the risk of disability.

Building on the above line of inquiry, we have adopted a more encompassing definition of multimorbidity (MM) by accounting for functional limitations and geriatric syndromes, rather than chronic conditions alone, thus drawing a clear distinction between MM and multiple chronic conditions. We have also shown that the co-occurrence of functional limitations and geriatric syndromes with chronic conditions is (i) highly prevalent in older adults, and (ii) has cumulative effects relative to health outcomes [20], thus reflecting greater disease burden. Such an encompassing approach is all the more relevant with the aging of the population, given that the findings will guide future developments in clinical care, research methodology, and healthcare policy. This is best reflected through the Department of Health and Human Services’ 2010 Strategic Framework on Multiple Chronic Conditions [21], which calls for studies to “*understand the epidemiology of multiple chronic conditions*” (Goal 4, Objective B), while highlighting the limited research about “*the constellations of conditions that are most prevalent and most important in terms of disability among individuals with MCC*.” This document further calls for additional research identifying the “*most common patterns of MCC,*” which can help in “*targeting specific interventions for specific subgroups and monitoring the impact of those interventions*.”

In the present study, we use rich survey data from the Health and Retirement Study (HRS) on a US representative sample of midlife and older adults to analyze the number of chronic conditions, functional limitations, and geriatric syndromes constituting MM across sociodemographic strata. We build upon our previous work [20] in two key ways. First, we provide a more detailed characterization of the profile of MM in midlife and older adults. Rather than examining the co-occurrence of chronic conditions, functional limitations, and geriatric syndromes in broad terms, we report on the most frequently co-occurring specific conditions constituting MM (CCMM). Second, in a subset of the study population receiving care through the Medicare FFS system, we analyze the number of CCMM in relation to Medicare expenditures. Given potential differences between younger (50–64 years of age) and older (≥65 years of age) Medicare beneficiaries in their MM patterns and expenditures, we conducted these analyses after stratifying the data by age.

The study hypotheses were: (i) older age is associated with a greater count of CCMM; (ii) a greater count of CCMM is associated with higher expenditures; (iii) a relatively smaller percentage of Medicare beneficiaries with a high count of CCMM incur a high percentage of Medicare expenditures; and (iv) rather than a large representation of HRS participants with specific combinations of CCMM, there are certain conditions, or combinations of conditions, that appear frequently with greater counts of CCMM.

## Objectives

The objectives of this study were to analyze patterns of CCMM and expenditures in a US representative sample of midlife and older adults (50–64 and ≥65 years of age, respectively).

## Methods

A cross-sectional study was performed using data from the 2010 HRS and linked Medicare data from 2010 to 2011. The study was approved by the University of Michigan, which administers the HRS; the Centers for Medicare & Medicaid Services (CMS) privacy board; and the Institutional Review Board at Case Western Reserve University.

### Data sources

#### The HRS

Launched in 1992, and supported by the National Institute on Aging, the HRS is a biennial survey of US representative sample of adults aged 50 years or older. To date, approximately 30,000 older adults have been surveyed by the HRS. In addition to sociodemographic variables, the HRS includes a broad range of variables on self-reported chronic conditions, functional status, cognitive status, and depressive symptoms, among others.

#### The Medicare beneficiary summary file (MBSF)

Individuals who agree to have their HRS survey data linked to their Medicare records can also be identified in Medicare administrative files. Thus, the MBSF was used to retrieve relevant data for HRS respondents who were Medicare beneficiaries, including those who enrolled in Medicare before age-qualifying for the program. The MBSF carries monthly variables indicating beneficiaries’ enrollment in Medicare managed care programs, making it possible to identify Medicare beneficiaries receiving care through the traditional system. The MBSF also includes the total Medicare expenditures incurred by FFS beneficiaries during a given calendar year. Thus, the MBSF was used to identify Medicare FFS beneficiaries and to analyze expenditures relative to the count of CCMM identified from the HRS.

#### The linked HRS–MBSF file

This cross-reference file, which is made available to researchers when accessing the restricted linked HRS–MBSF files, enables portions of the records from each of the HRS and MBSF to be linked and to construct a unique record for each individual in the study population.

### Study population

A total of 18,005 individuals were surveyed in 2010. This did not include proxy respondents or former HRS participants who died prior to the 2010 scheduled interview. Records for individuals with missing data on study variables (*n*=93) were excluded, leaving a total of 17,912 in the study population.

As noted above, Medicare data were obtained from the MBSF for 5,677 individuals (*n*=648 for 50–64-year olds and *n*=5,029 for ≥65-year olds) who were enrolled in Medicare, regardless of their age, and who had received their care services through the FFS program.

### Key variables of interest

#### Multimorbidity

Variables included in our definition of MM were not only self-reported chronic conditions but also functional limitations and geriatric syndromes. The following specific conditions were accounted for in each of the aforementioned broad rubrics:

- Self-reported chronic conditions (possible total count of eight), indicating whether the individual was ever told by a physician that he or she had hypertension, arthritis, heart disease, lung disease, non-skin cancer, stroke, diabetes, and psychiatric conditions.
- Functional limitations (possible count of five): upper body limitations (e.g. difficulty picking up a coin from the floor or reaching overhead); lower body limitations (e.g. difficulty climbing stairs or walking around the block); strength limitations (e.g. ability to lift 4.5 kg or difficulty in moving a large object); limitations in activities of daily living (ADL; e.g. crossing a room, dressing, bathing); and limitations in instrumental ADL (IADL; e.g. ability to prepare meals or manage money).
- Geriatric syndromes (possible count of seven): poor cognitive functioning (lowest tertile of cognitive score obtained from the Telephone Interview Cognitive Survey [TIC]); depressive symptoms (answered “yes” to at least four of the eight items of the Center for Epidemiological Studies-Depression [CES-D] scale); visual impairment; hearing impairment; often experience severe pain; incontinence; and dizziness.

Thus, the total count of conditions that an individual may potentially present with is 20.

#### Annualized median Medicare expenditures

The MBSF includes the total amount of Medicare expenditure incurred by a beneficiary in a given year. To account for partial-year enrollment caused by entering mid-year or exiting the program (mainly through death), we calculated the per-person per month (PPPM) expenditures by aggregating the Medicare expenditures in 2010 and 2011, and dividing the total amount by the total number of FFS months during the 2-year period. To obtain more robust estimates, we calculated the PPPM based on 2-year rather than 1-year data. To annualize, we then multiplied the PPPM expenditures by 12, and afterwards by 0.01 to account for inflation from 2010 to 2011. Data are presented as median to address the skewedness of expenditures.

### Other variables of interest

Other data retrieved from the HRS included age (<64, 65–74, 75–74, and ≥85 years); sex; race (Non-Hispanic White, Non-Hispanic Black, Hispanic, and other); marital status (married and non-married); years of education (<9, 9–11, 12, 13–15, 16, and ≥17); income (<100% of the federal poverty level, 100–199%, 200–299%, and ≥300%); and self-reported Medicaid receipt (yes/no).

### Analysis

Using survey weights to account for the complex survey design of the HRS, we conducted a descriptive analysis to identify patterns of CCMM count in the sociodemographic strata of our study population and to report the median annualized expenditures for CCMM. To analyze the combinations of CCMM, we used two different approaches. First, we used association rule mining (ARM) to identify the most common monads, dyads, triads, quadriads, and pentads, and to determine whether or not they co-occur with any other conditions. ARM was developed in the realm of marketing research to identify items that are commonly purchased together and is now applied in a variety of settings, including medicine and bioinformatics [22–24]. Second, we identified the five most frequently observed specific CCMM that were identified in each of the count categories. For parsimony, we limited our reporting of CCMM to the top five most-frequently observed combinations. We used R version 3.3.1 and “arules” package 1.4-1 for the ARM analysis, and SAS version 9.3 for all other analyses.

## Results

Of the 17,912 HRS participants included in our study, about half (50.9%) were younger than 65 years of age, 42.7% were men, 31.3% were non-Hispanic Black or Hispanic, 21% had less than 12 years of education, 32.3% had an income of less than 200% of the federal poverty level, and 6.2% were on Medicaid or dually eligible Medicare–Medicaid beneficiaries (Table 1).

Only 15.6% of study participants presented with no or only one condition, and 22.3% presented with two or three conditions. The remaining 62% of the population presented with at least four conditions, including 11.4% with 10 or more conditions.

A higher count of conditions was observed among older than younger individuals. For example, 7.4% of individuals aged 50–64 years presented with 10 or more conditions, compared with 31.8% of those aged ≥85 years. Similarly, higher counts were observed among women than among men; among those with lower income and/or educational attainment than among those with higher income and/or educational attainment; and among those on Medicaid or dually eligible Medicare–Medicaid beneficiaries (Table 1).

Our analysis of the combinations of conditions is described below, and is as follows: first, we show *the most common combinations of conditions* (monads, dyads, etc.) that appear regardless of whether or not they co-occur with other conditions (e.g. combination of hypertension and arthritis, with or without the co-occurrence of strength limitations or incontinence); next, we show the number of *individuals with specific combinations* *of conditions*, without the co-occurrence of other conditions; and lastly, we present results showing the association between the number of conditions with which individuals present and the Medicare expenditures.

The most common *monads, dyads, triads, quadriads, and pentads* of combinations within CCMMs are shown in Tables 2A (age 50–64 years) and 2B (age ≥65 years). The conditions are color-shaded to highlight the frequency with which certain conditions appear across the different count categories.

Among individuals aged 50–64 years, the most common condition was hypertension (50.0%), whether presenting with or without the co-occurrence of any other condition (Table 2A). The most common dyad was limitations in lower body functioning and strength limitations (37.2%). For triads, the most common conditions were hypertension, limitations in lower body functioning, and strength limitations (24.0%). The most common quadriad consisted of hypertension, arthritis, limitations in lower body functioning, and strength limitations (16.4%). The most common pentad included the aforementioned conditions, in addition to limitations in upper body functioning (11.0%) (Table 2A).

Among individuals aged ≥65 years, the most frequent monads, dyads, triads, quadriads, and pentads were identical to those observed in the younger age group (50–64 years), but the percent of individuals presenting with these combinations of conditions differed markedly (Tables 2A and 2B). For example, 72.3% of adults aged ≥65 years had hypertension compared with 50.0% of those in the younger age group, whether or not hypertension co-occurred with any other condition. Similarly, 25.3% of individuals aged ≥65 years presented with the pentad of hypertension, arthritis, limitations in upper body functioning, limitations in lower body functioning, and strength limitations, compared with only 11.0% of those in the younger age group. Interestingly, the most common monad, dyad, quadriad, and pentad appearing in combinations of conditions was identical across the two age groups.

The percent of *individuals* presenting with the top five most frequent combinations of CCMM is shown in Tables 3A (age 50–64 years) and 3B (age ≥65 years). As noted above, this analysis differs from the one presented in Tables 2A and 2B in that it accounts for the exact combinations of conditions with which individuals present, without the co-occurrence of other conditions. Again, for parsimony, only combinations up to a count of five are shown.

In both age groups of the population, our analysis did not show any large representations of HRS participants with specific CCMM (Tables 3A and 3B). Rather, we observed great heterogeneity in the grouping of individuals with specific combinations of CCMM. For example, for individuals aged 50–64 years, the most frequent CCMM for two conditions (hypertension and poor cognition) accounted for only 96 of the 1,422 individuals (6.8%) presenting with two conditions (Table 3A). Similarly, for individuals aged ≥65 years, the most frequent CCMM for two conditions (hypertension and arthritis) accounted for 80 of the 620 individuals (12.9%) presenting with this combination of conditions (Table 3B). Of note, the combination of hypertension and arthritis was also frequently observed in individuals of both age groups with CCMM counts greater than two. Moreover, we note the prominence of functional limitations in these combinations of conditions, especially lower body limitations and strength limitations. In particular, the combination of limitations in lower body functioning and strength limitations begins to emerge – and is consistently present – among individuals presenting with a CCMM count of three or greater.

**Figure 1:**
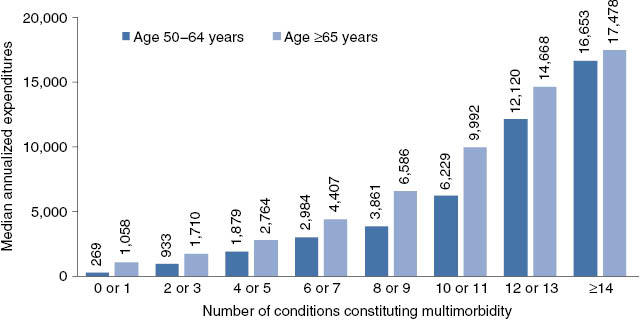
Median annualized expenditures by total number of conditions constituting multimorbidity, stratified by age (Medicare fee-for-service beneficiaries only).

Figure 1 shows expenditure data related to counts of CCMM, stratified by age group. These analyses are limited to HRS participants who are also Medicare beneficiaries, and receive their care through the FFS program; hence, the distribution of individuals by the CCMM is different from that described above for all HRS participants. The median annualized expenditures increases considerably with the number of CCMM with which individuals present. However, rather than a straight line, the association is “J-shaped,” showing a sharp increase in the expenditures with greater CCMM counts.

**Figure 2:**
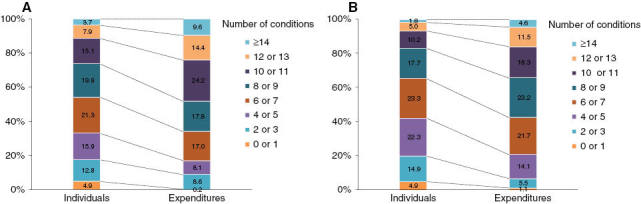
Distribution of individuals (Medicare fee-for-service beneficiaries only) and expenditures by total number of conditions constituting multimorbidity: (A) age 50–64 years (*n*=648), (B) age ≥65 years (*n*=5,029).

The distribution of individuals and expenditures by CCMM, and stratified by age, is shown in Figures 2A and 2B. In individuals aged 50–64 years, 26.7% of the participants presented with 10 or more CCMM, but incurred almost half (48.2%) of the expenditures (Figure 2A). In those aged ≥65 years, 17.0% presented with 10 or more CCMM and incurred 34.4% of the expenditures (Figure 2B).

## Discussion

Using a unique resource combining survey and administrative data for a representative sample of US midlife and older adults, we evaluated the MM burden by examining the count of CCMM across sociodemographic strata, as well as in terms of Medicare expenditures. We conducted this analysis using data for all HRS participants, as well as on the subset of HRS participants who are also Medicare FFS beneficiaries. Our findings indicate that functional limitations and geriatric syndromes considerably add to the MM burden in midlife and older adults, and that expenditures increase sharply with higher counts of CCMM.

The disproportionate consumption of Medicare expenditure by individuals with multiple chronic conditions has been reported previously, though the extent of the disproportionality varies across different sources [25–27]. In a Robert Wood Johnson Foundation chartbook, Anderson reported that two thirds of the Medicare expenditure is incurred by individuals with five or more chronic conditions [25], while data from the CMS indicate that 15% of Medicare FFS beneficiaries present with six or more chronic conditions, but incur 51% of the Medicare expenditures [26]. While our measures are not directly comparable to the ones used in the aforementioned reports, we note that by incorporating functional limitations and geriatric syndromes in our count of CCMM, we demonstrate a considerably greater burden associated with MM: among Medicare beneficiaries aged 50–64 years, 68% presented with six or more CCMM and incurred 83% of the Medicare expenditures. Among beneficiaries aged ≥65 years, these percentages were 58% and 79%, respectively. Our findings show that a greater count of CCMM is associated with higher expenditures, possibly as a result of co-occurring functional limitations and geriatric syndromes. The contribution of functional limitations to higher expenditure is supported by our more recent study [28] in which 64% of individuals aged ≥65 years with self-rated poor health, as well as limitations in ADL and IADL, incurred expenditures in the top quartile, highlighting the importance of functional limitations in explaining resource use. We note, however, that in our current study, even our statistics may underestimate the burden of MM, given that we limited our list of chronic conditions to the eight self-reported in the HRS. The burden would be considerably higher if our algorithm relied on a broader range of chronic conditions identified from claims data.

Despite the difference in the study measures, the high burden of MM shown in our current analysis is somewhat consistent with a report by the Agency for Healthcare Research and Quality (AHRQ), indicating that 80% of individuals aged ≥65 years have multiple chronic conditions [27], using data from the Medical Expenditure Panel Survey, and Hwang’s definition of chronic condition as “*one that lasted or was expected to last twelve or more months and resulted in functional limitations, and/or the need for ongoing medical care*” [29]. Data from the AHRQ report also indicate that 45% of individuals with multiple chronic conditions have functional limitations, including ADL, IADL, as well as any functional, activity, or sensory limitations [28].

Our results are also comparable to that of a recent study by McClintock *et al*. [30] who assessed population health based on a Comprehensive Model, which, in contrast to the Medical Model, incorporates elements of health as defined by the World Health Organization (e.g. mental health). Using data from the National Social Life, Health, and Aging Project, and latent class analysis, the Comprehensive Model identified six health classes, including two classes, in which mental health (loneliness), hearing impairment, and bone fractures emerged as important conditions in defining vulnerable health classes. In addition, whereas the Medical Model grouped two thirds of the population into “robust health” classes, the Comprehensive Model classified half of the population as having significant vulnerabilities that would impact their health outcomes.

Regarding CCMM, our findings failed to identify large groups of individuals with specific combinations of CCMMs (Tables 3A and 3B), but did identify combinations of CCMMs that co-occur frequently (Tables 2A and 2B). This attests to the heterogeneity of individuals with MM or multiple chronic conditions, as reported by Whitson *et al*. [31]. A notable finding, however, is that in individuals with two or more CCMM, we begin observing the prominence of functional limitations and geriatric syndromes. In particular, functional limitations are almost always present in individuals presenting with three or more CCMM. In addition, the combination of limitations of lower body functioning and strength limitations are the most frequently observed dyad, regardless of whether they co-occur with other conditions, presenting in 37.2% of individuals aged 50–64 years of age, and in 57.5% of individuals aged ≥65 years.

These results have important implications both in research and in clinical practice, warranting a shift of paradigm to account for functional limitations and geriatric syndromes when studying MM and not simply focusing on chronic conditions. In addition to being closely inter-related [32–35], the former conditions also have important implications relative to health and healthcare outcomes [36–40], health services utilization, including hospitalizations and readmissions [41,42], as well as to clinical practice and healthcare policy. First, we believe that these data strongly support the notion that MM in midlife and older adults is the norm, rather than the exception, and – given the heterogeneous combinations of the various conditions that we identify – that the care they receive must be person-centered, rather than disease/condition-centered. Second, our findings call for the wide adoption of such instruments as the Comprehensive Geriatric Assessment [43] to routinely evaluate patients’ physical, mental, affective, and sensory functioning, and to identify subgroups of the midlife and older adults who are most vulnerable to experience adverse outcomes. Similarly, these findings call for the expansion and broader adoption of such programs as *Independence at Home* [44], authorized by the Affordable Care Act to provide primary care home visits to individuals with multiple chronic conditions and functional limitations. This demonstration program, aimed at improving patient and caregiver satisfaction and reducing the need for hospitalization, has yielded savings of over USD 1,000 per beneficiary [44]. However, it is important that such programs also place emphasis on geriatric syndromes for eligibility criteria. By doing so, the program would have a considerably greater impact relative to prevention as well. For example, poor cognitive performance alone may not qualify an individual for such a program; yet, it is listed as one of the most frequent conditions, and part of the most common dyads that also includes hypertension. Based on the circular association between chronic conditions and geriatric syndromes described earlier, a person with hypertension who also has cognitive impairment is likely to suffer poor self-management, therefore poor blood pressure control and complications. Finally, our findings call for the availability of multidisciplinary care teams, and healthcare providers who can help community-dwelling midlife and older adults, not only with the management of their chronic conditions but also with their functional, cognitive, and sensory impairments. Such assistance may be paramount to helping individuals stay at home and delay, or even prevent institutionalization.

Findings from this study also have implications regarding MM burden in midlife adults. Indeed, as shown in Table 1, nearly 28% of those aged 50–64 years have ≥6 CCMMs. These statistics inform us of the anticipated MM burden in incoming cohorts of Medicare beneficiaries.

In conclusion, the prominence of co-occurring functional limitations and geriatric syndromes in CCMMs provides a basis for developing clinical systems that consider the interaction among co-existing conditions and treatments, and the needed resource allocation. Studies on MM bear increasingly greater significance, especially given the aging of the population, as their findings will guide future developments in clinical care for older adults, research methodology, and policy analysis.

## Figures and Tables

**Table 1:** Distribution of the study population by sociodemographic characteristics and by number of conditions constituting multimorbidity.

	Number of chronic conditions, functional limitations, and geriatric syndromes						
	*N* (% of total study population)	0 or 1 *N* (%)	2 or 3 *N* (%)	4 or 5 *N* (%)	6 or 7 *N* (%)	8 or 9 *N* (%)	≥10 *N* (%)
Total population	17,912 (100.0)	2,802 (15.6)	4,000 (22.3)	3,743 (20.9)	3,200 (17.9)	2,129 (11.9)	2,038 (11.4)
Age, years							
50–64	9,111 (50.9)	2,285 (25.1)	2,554 (28.0)	1,751 (19.2)	1,170 (12.8)	680 (7.5)	671 (7.4)
65–74	4,254 (23.7)	391 (9.2)	929 (21.8)	1,086 (25.5)	938 (22.0)	499 (11.7)	411 (9.7)
75–84	3,246 (18.1)	112 (3.5)	443 (13.6)	734 (22.6)	808 (24.9)	607 (18.7)	542 (16.7)
≥85	1,301 (7.3)	14 (1.1)	74 (5.7)	172 (13.2)	284 (21.8)	343 (26.4)	414 (31.8)
Sex							
Male	7,640 (42.7)	1,439 (18.8)	1,897 (24.8)	1,610 (21.1)	1,262 (16.5)	765 (10.0)	667 (8.7)
Female	10,272 (57.3)	1,363 (13.3)	2,103 (20.5)	2,133 (20.8)	1,938 (18.9)	1,364 (13.3)	1,371 (13.3)
Race/ethnicity							
White Non-Hispanic	11,770 (65.7)	1,881 (16.0)	2,621 (22.3)	2,544 (21.6)	2,138 (18.2)	1,388 (11.8)	1,198 (10.2)
Black Non-Hispanic	3,346 (18.7)	439 (13.1)	706 (21.1)	683 (20.4)	601 (18.0)	413 (12.3)	504 (15.1)
Hispanic	2,264 (12.6)	356 (15.7)	524 (23.1)	434 (19.2)	379 (16.7)	280 (12.4)	291 (12.9)
Other	532 (3.0)	126 (23.7)	149 (28.0)	82 (15.4)	82 (15.4)	48 (9.0)	45 (8.5)
Marital status							
Married	7,250 (40.5)	822 (11.3)	1,339 (18.5)	1,382 (19.1)	1,377 (19.0)	1,090 (15.0)	1,240 (17.1)
Not married	10,662 (59.5)	1,980 (18.6)	2,661 (25.0)	2,361 (22.1)	1,823 (17.1)	1,039 (9.7)	798 (7.5)
Education, years							
<9	1,696 (9.5)	98 (5.8)	249 (14.7)	296 (17.5)	339 (20.0)	306 (18.0)	408 (24.1)
9–11	2,068 (11.5)	133 (6.4)	298 (14.4)	408 (19.7)	447 (21.6)	376 (18.2)	406 (19.6)
12	5,638 (31.5)	700 (12.4)	1,178 (20.9)	1,224 (21.7)	1,172 (20.8)	717 (12.7)	647 (11.5)
13–15	4,256 (23.8)	752 (17.7)	1,044 (24.5)	944 (22.2)	702 (16.5)	432 (10.2)	382 (9.0)
16	2,210 (12.3)	579 (26.2)	642 (29.0)	438 (19.8)	278 (12.6)	167 (7.6)	106 (4.8)
≥17	2,044 (11.4)	540 (26.4)	589 (28.8)	433 (21.2)	262 (12.8)	131 (6.4)	89 (4.4)
Income as % of federal poverty level							
<100	2,315 (12.9)	183 (7.9)	355 (15.3)	447 (19.3)	416 (18.0)	376 (16.2)	538 (23.2)
100–199	3,479 (19.4)	269 (7.7)	514 (14.8)	643 (18.5)	769 (22.1)	625 (18.0)	659 (18.9)
200–299	2,954 (16.5)	333 (11.3)	590 (20.0)	638 (21.6)	617 (20.9)	427 (14.5)	349 (11.8)
≥300	9,164 (51.2)	2,017 (22.0)	2,541 (27.7)	2,015 (22.0)	1,398 (15.3)	701 (7.6)	492 (5.4)
Medicaid status							
No	16,796 (93.8)	2,784 (16.6)	3,935 (23.4)	3,598 (21.4)	2,995 (17.8)	1,872 (11.1)	1,612 (9.6)
Yes	1,116 (6.2)	18 (1.6)	65 (5.8)	145 (13.0)	205 (18.4)	257 (23.0)	426 (38.2)

**Table 2A:**
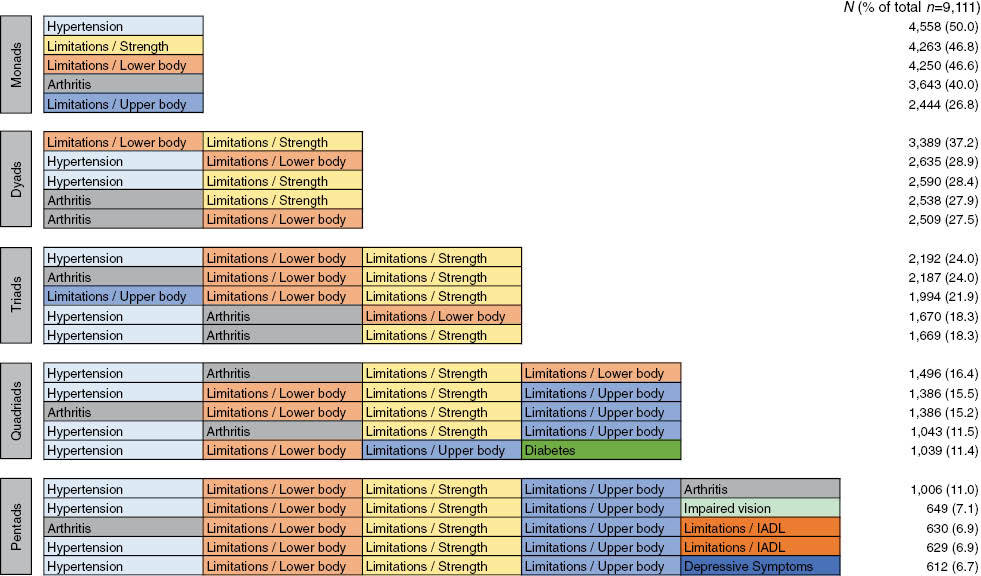
Five most frequently observed monads, dyads, triads, quadriads, and pentads appearing in combinations of conditions constituting multimorbidity among adults aged 50–64 years.

IADL, instrumental activities of daily living.

**Table 2B:**
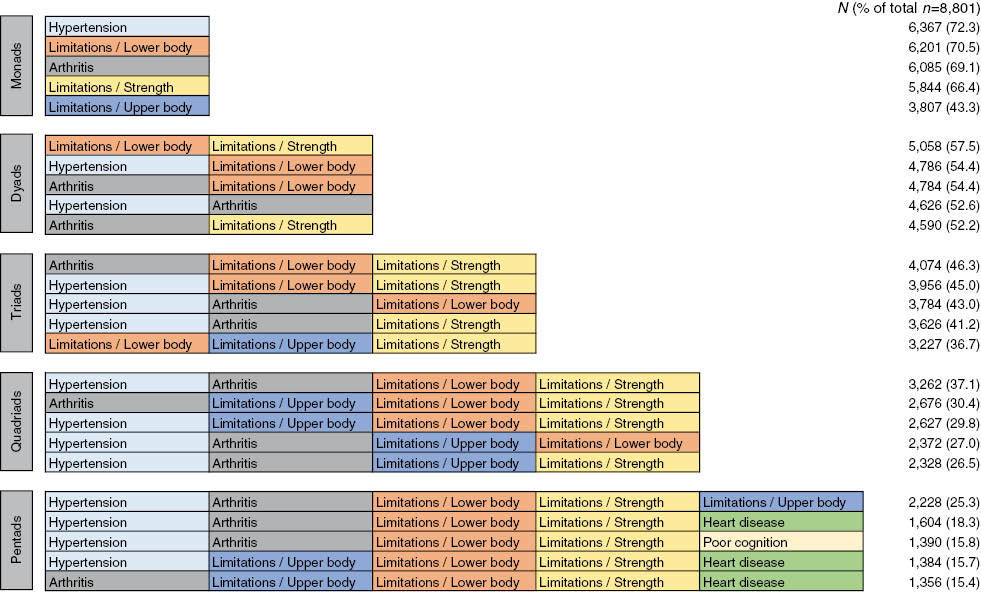
Five most frequently observed monads, dyads, triads, quadriads, and pentads appearing in combinations of conditions constituting multimorbidity among adults aged ≥65 years.

**Table 3A:**
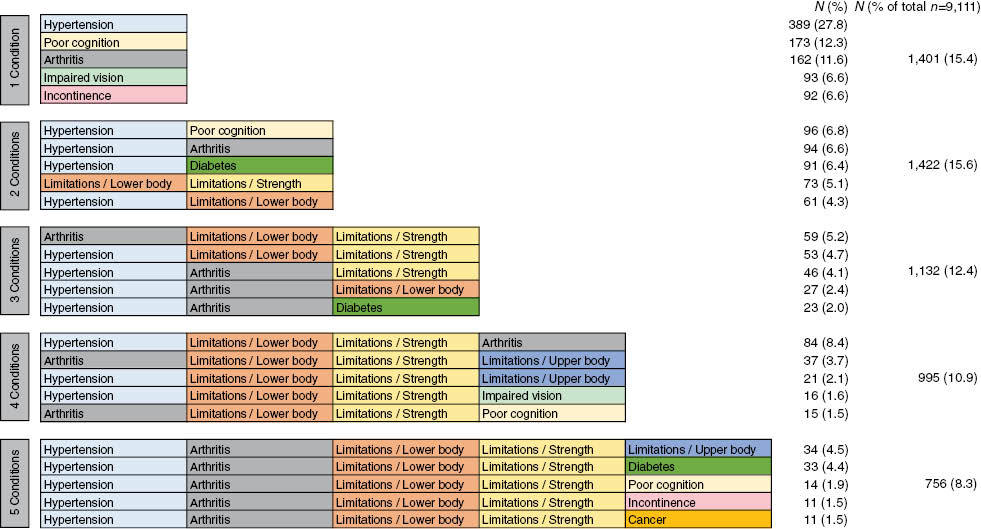
Most frequent combinations of conditions constituting multimorbidity among adults aged 50–64 years, by number of conditions (top five only).

**Table 3B:**
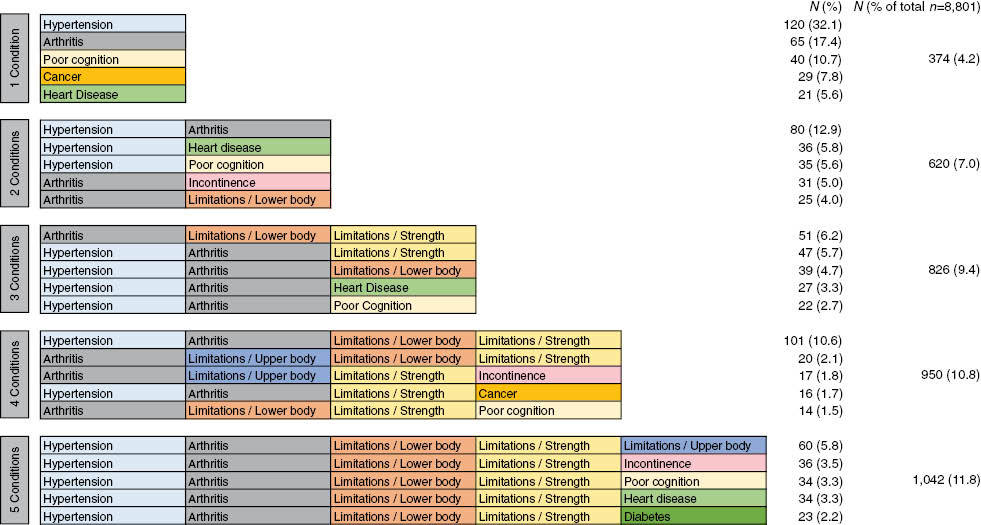
Most frequent combinations of conditions constituting multimorbidity among adults aged ≥65 years, by number of conditions (top five only).
